# Post-traumatic severe ankle valgus and forefoot supination deformities treated by staged surgery using ilizarov technique and limited osteotomy: A case report

**DOI:** 10.1097/MD.0000000000028385

**Published:** 2021-12-23

**Authors:** Jie Zhang, Yongmei Li, Luping Liu, Leijie Chen, Zhou Liu, Qihui Duan, Bo Pu

**Affiliations:** a Orthopedics Department, The 2nd Affiliated Hospital of Kunming Medical University, Kunming, Yunnan, China; b Rehabilitation Department, The 2nd Affiliated Hospital of Kunming Medical University, Kunming, Yunnan, China.

**Keywords:** ankle valgus deformity, forefoot supination deformity, ilizarov external fixation, osteotomy, scar contracture

## Abstract

**Rationale:**

Post-traumatic ankle valgus deformities are relatively rare. Old post-traumatic ankle deformity compounded by abundant scar contracture tissue formation around the joint is a big challenge for orthopedics. Conventional one-stage corrective osteotomy with internal fixation always results in many knotty postoperative complications, such as soft tissue avascular necrosis, implant-related infections, and distinct lower limb discrepancy. Here, we describe a patient with old post-traumatic severe ankle valgus and forefoot supination deformities and obtained satisfactory clinical results following multi-stage surgery using the Ilizarov technique and limited osteotomy. Even more encouraging, any complications of conventional one-stage surgery were successfully avoided through our treatment regimen.

**Patient concerns:**

A 24-year-old healthy man had post-traumatic 90-degree hindfoot valgus and forefoot supination deformities of the right foot for more than 10 years. The complicated issue was the vast, poorly vascularized scar contracture tissues tightly adhered to the bones of the lateral malleolus and dorsum pedis.

**Diagnoses:**

Old post-traumatic severe ankle valgus and forefoot supination deformities and scar contracture of soft tissues of the foot and ankle joint.

**Interventions:**

In the first stage, Ilizarov external fixation was used to stretch the scar contracture tissue of the lateral malleolus. In the second stage, limited osteotomy of the tibiotalar joint and progressive closure of the osteotomy site were performed. In the third stage, Chopart joint osteotomy and slow forefoot pronation by external frame were performed.

**Outcomes:**

Our treatment regimen not only guaranteed soft tissue safety, but also avoided infection and obvious lower limb discrepancy. At the 1-year follow-up, the patient acquired aesthetic and functional right foot.

**Lessons:**

Although relatively rare, old post-traumatic severe ankle valgus and forefoot supination deformities can be corrected using Ilizarov external fixation technology combined with limited osteotomy. With a well-designed staged operation scheme, soft tissue avascular necrosis, infection of the wound, obvious lower limb discrepancy, and flap grafting can be avoided.

## 1 Introduction

Secondary deformities of the foot and ankle are mostly due to inappropriate initial treatments of serious traumatic injuries, such as traffic accidents that lead to malunion or severe burns, resulting in the formation of cicatrices and chronic deformities. Traditional triple arthrodesis mainly involves a single-stage surgical procedure that includes releasing the surrounding soft tissues, and multi-site osteotomies of the calcaneus, talus, and cuboid bones to achieve deformity corrections.^[1,2]^ This surgery causes shrinkage of the affected foot by eliminating many bone masses. In addition, the wide debonding and resection of cicatricial tissues generate wound necrosis, and secondary skin flap grafting is often required.^[3,4]^ Moreover, the effective blood supply to the affected area is significantly decreased and the defense barrier is destroyed due to the extensive exposure and release of the bones and soft tissues, which not only increases the risk of infection but also makes wound healing more difficult.^[5]^ Along with the acute traction of the neurovascular bundle, which jeopardizes the safety of soft tissues during this one-stage corrective maneuver, there are many complications such as postoperative joint stiffness and pseudo-articular formation.^[6]^

In recent years, the technique of annular external fixation based on the principle of Ilizarov technology has made good progress in the clinical treatment of equinus, cavus, calcaneus deformity, and anterior foot adductus or valgus.^[7]^ Compared with traditional single-stage surgery, this multi-stage scheme provides more soft tissue safety and less osteotomy. We report a case of severe post-traumatic ankle valgus and forefoot supination deformities treated by staged surgery using the ilizarov technique and limited osteotomy.

## 2 Case report

**Figure 1:**
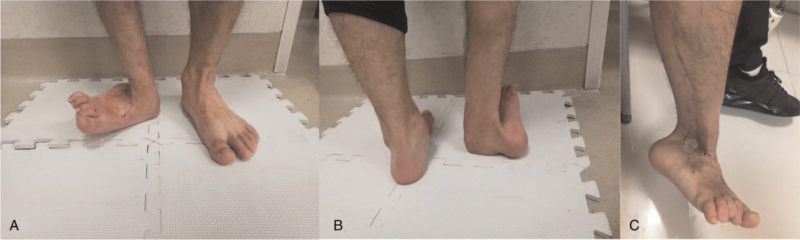
Appearance before operation. (A and B) the right hindfoot was 90-degeree valgus deformity, and the weight-bearing area had been transferred from the calcaneus to the distal end of the tibial. (C) Abundant scar contracture tissues tightly adhered to the bones of the lateral malleolus and the dorsum pedis.

A 24-year-old otherwise healthy man was admitted to the hospital for more than 10 years with a traumatic right foot deformity. Physical examination revealed that the right hindfoot was 90-degree valgus together with ankle joint stiffness, and the weight-bearing part was the distal end of the right tibia (Fig. 1A, B). Abundant scar contracture tissues tightly adhered to the bone were observed in the area of the lateral malleolus and foot dorsum (Fig. 1C). The range of movement of the ankle was limited to 15°of dorsiflexion without plantar flexion. Except for the V-grade tibialis anterior muscle, the rest of the muscle strength could not be examined. In addition to hypoesthesia, the dorsal artery of the foot cannot be felt.

**Figure 2:**
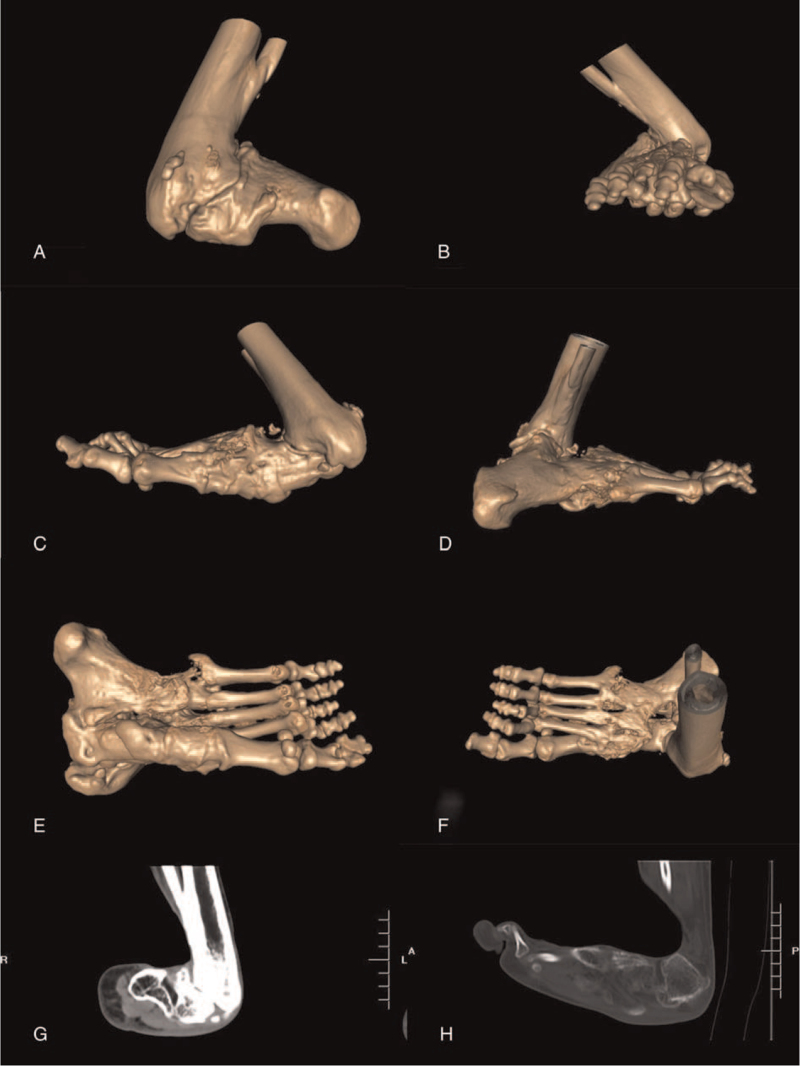
CT scan and 3-D reconstruction images before operation. (A–F) The lateral malleolus was disappeared and the inferior tibiofibular joint was deformed fusion. Tarsometatarsal, talocalcaneonavicular, talocalcaneal and calcaneocuboid joints were bony fusion. The arch of the foot was collapsed. (G and H) Inferolateral misdirection of tibiotalar joint surface and abnormal shapes of tarsal bones, calcaneus and talus.

Computed tomography and three-dimensional reconstruction showed the right foot tarsometatarsal joint, talocalcaneonavicular joint, talocalcaneal joint, calcaneocuboid joint, and inferior tibiofibular joint were stiff and deformed fusion. The normal shapes of the right foot tarsal bones, calcaneus, and talus disappeared. With the disappearance of the lateral malleolus, the talus turned outward and upward, forming a distortional joint with the tibia. The arch of the right foot collapsed (Fig. 2).

**Figure 3:**
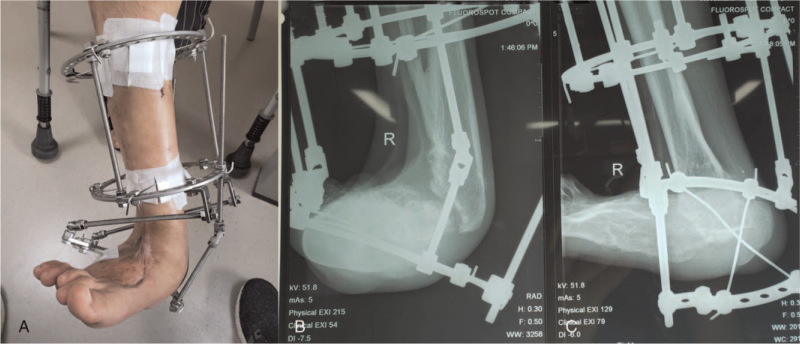
Appearance and X-ray images after the first-stage operation. (A) The ankle joint was in situ fixed by Ilizarov annular external fixation system. A contraction adjusting rod was connected to the medial part of the semicircular brace. (B and C) The semicircular brace was connected to the calcaneus by 2 cross Kirschner wire.

**Figure 4:**
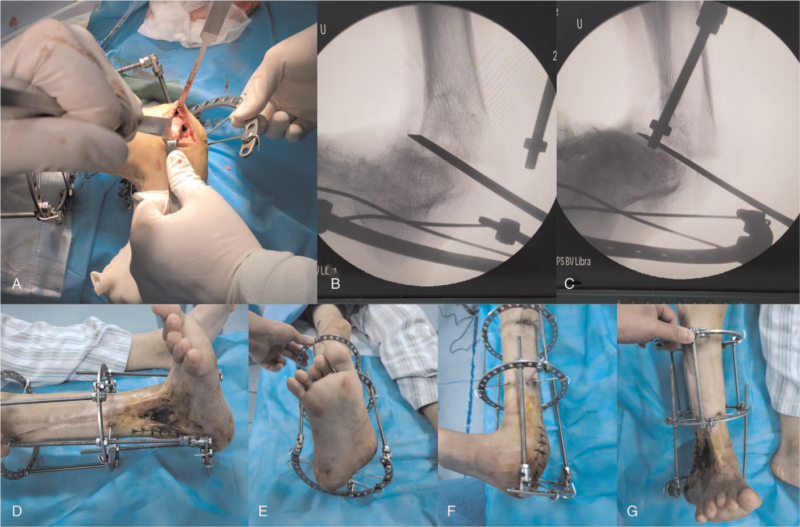
Limited osteotomy of the hindfoot during the second-stage operation. (A, B and C) At the medial side of tibiotalar joint, about 45-degress wedge-shaped bone was removed. (D) A 5 cm longitudinal skin incision was made for limited releasing of the lateral malleolus scar tissues. (E, F and G) The hindfoot was maintained at its valgus site without intentional closure of the osteotomy gap.

**Figure 5:**
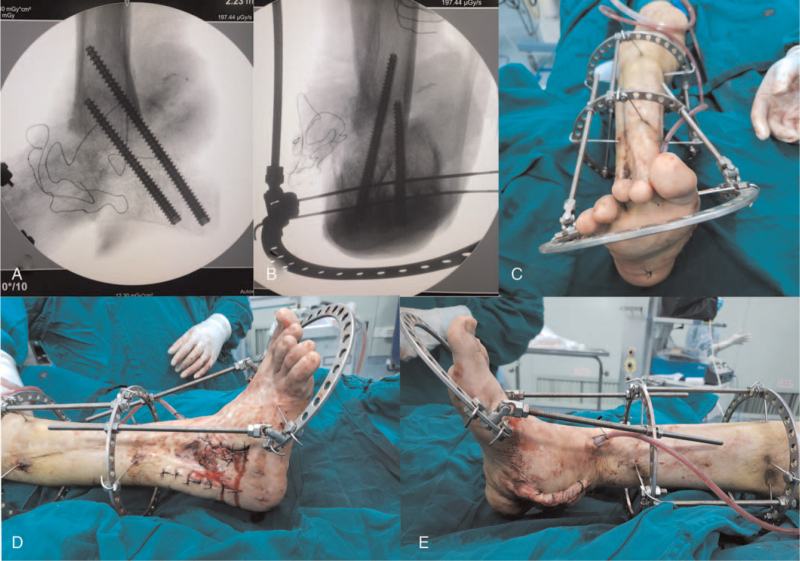
The hindfoot internal fixation and the forefoot external fixation during the third-stage operation. (A and B) When the hindfoot got neutral position, the previous external semicircular brace was removed and 2 countersunk tension screws were used to internally fix the hindfoot. (C, D and E) Bony fusions of talonavicular and calcaneocuboid joints were cut by osteotome through medial and lateral approaches, respectively. The supine forefoot was in situ fixed by a three-fourth ring brace which had the medial and the lateral adjusting rods connecting to other part of Ilizarov annular external fixation system.

The patient underwent his first operation after admission to the hospital. According to the Ilizarov principle, an annular external fixation system was adopted to fix the ankle joint in situ. A contraction-adjusting rod was connected to the medial part of the semicircular brace, which controls the calcaneus using two cross Kirschner wires (Fig. 3). In an attempt to stretch the scar tissue of the lateral malleolus, the initial rate of contraction was 1 mm per day. With growing pain at the lateral part of the ankle, this procedure was stopped at 3 months postoperatively, and 30-degree reducing of hindfoot valgus was achieved. Aiming to place the calcaneus from valgus to neutral position, the second-stage osteotomy surgery was performed to remove a wedge-shaped bone at the medial side of the tibiotalar joint (Fig. 4AB, and C). However, direct end closure of the osteotomy site could still not be obtained due to the great tension of the lateral malleolus cicatricial contracture. Then a 5 cm longitudinal surgical incision was made at the lateral malleolus to limit the release of scar tissue (Fig. 4D). The hindfoot was maintained at its valgus site by the original annular external fixation, but without intentional closure of the wedge-shaped gap of the osteotomy site (Fig. 4E, F, and G). After 2 weeks of slow traction at a speed of 1 mm per day, the hindfoot was placed neutrally, and the third stage operation was administered to the patient. With 2 countersunk tension screws to fix the hindfoot percutaneously, the semicircular brace of the calcaneus was removed (Fig. 5AB). Since the forefoot was at supination position when the hindfoot was neutral, deformative fused talonavicular and calcaneocuboid joints were cut by an osteotome, and the metatarsal bones were connected to a three-fourth-ring brace by 2 Kirschner wires (Fig. 5C, D, and E). By regulating the medial and lateral adjusting rods at a speed of 1 mm per day, the forefoot was turned to a neutral position for 3 weeks. One month after the third operation, the patient was allowed to ambulate with partial weight bearing for 2 months, after which the annular external fixation system was removed in the clinic.

**Figure 6:**
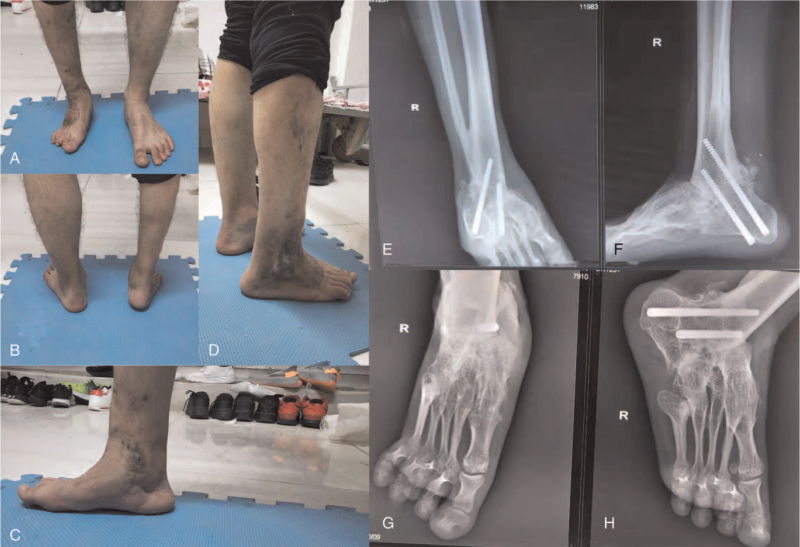
One year follow-up appearance and X-ray images. (A, B) The valgus of the hindfoot and the supination of the forefoot were corrected. (C) The arch of the foot was restored. (D) The cicatricial tissues of the lateral malleolus and the dorsum pedis were safely preserved. (E–H) All osteotomy sites got bony union.

One year follow-up suggested that the hindfoot valgus and forefoot supination deformities were corrected, the arch of the right foot was restored, and all soft tissues around the ankle were safely preserved (Fig. 6A–D). The three-point weight-bearing model of the right foot, namely the first metatarsal head, fifth metatarsal head, and calcaneus, was restored. Although a 1.5 cm shortening of the right lower limb was detected, it was clinically acceptable. X-ray revealed bony union at all osteotomy sites (Fig. 6E–F). The preoperative American Orthopedic Foot and Ankle Society ankle-hindfoot score was 9 points compared with the postoperative score of 73 points.

## 3 Discussion

Ilizarov external fixation is a mature technology that is widely used in orthopedics to treat complex fractures, severe limb injuries, limb functional reconstruction, and correction of congenital or traumatic deformities.^[8]^ By applying a sustained tension to the deforming force, the Ilizarov external fixation technique enables the formation of the deformities to be reversed. That is, under sustained tension force, the contracture scar tissues and muscles around the joint are gradually prolonged without ischemic necrosis, which reduces the severity of the deformity. Reduce the extent of secondary surgical resection and the difficulty of surgery

In this case, the biggest challenge was the vast, poorly vascularized scar contracture tissues tightly adhered to the bones of the lateral malleolus and dorsum pedis. Extensive release of these tissues must lead to avascular necrosis and the need for coverage by the pedicle vascular flap. Additionally, considering the 90-degree valgus deformity of the hindfoot, a large piece of wedge-shaped bone had to be removed to achieve correction by the conventional one-stage osteotomy and internal fixation surgery, which could cause obvious discrepancies in the lower limbs. Moreover, conventional plates and screws covered by insufficient blood supply soft tissues would make the operative site at a high risk of infection. Instead, we chose Ilizarov external fixation to gradually extend the lateral malleolus in the first-stage surgery, which not only guaranteed the safety of the scar tissues but also alleviated the severity of deformity to lessen osteotomy in the second-stage surgery. In addition to addressing the problem of blood supply to the cicatricial tissues, after the osteotomy operations in the second and the third stage of surgery, respectively, osseous deformity corrections were still slowly completed by Ilizarov external fixation, which provided sufficient stability for bone fusion and not only reduced or avoided implant use but minimized the infection risk. To avoid deformity recurrence during weight bearing, external fixation was left for 2 months before final removal. Compared with the previous long-term fixation of plaster, it reduced the skin necrosis caused by plaster pressing on the unhealthy skin.

Ilizarov external fixation and sural neurocutaneous flaps were used for one-stage reconstruction of contracture ankle deformity.^[9]^ The shortcoming is that the bloated ankle site influenced shoe wearing and joint motion. In our work, the multi-stage traction scheme combining limited osteotomy is a crucial part of avoiding flap use. However, it still needs to pay attention to several points^[10–12]^:

1. the process of stretching or pulling must be slow, in order to reduce the pain of the patient, achieve effective prolongation, and avoid local necrosis for vascular injury or new fracture due to excessive traction.
2. After the operation, the patient should walk with a stick and load gradually, and the annular external fixator should be left until effective bone fusion to avoid or reduce the deformity rebound.
3. as it takes a long time to wear external fixation, attention should be paid to disinfection around the nail channel to avoid infection or skin necrosis around the nail channel.
4. the extent of osteotomy and the length of the final stretch should be reasonably balanced, and bone transport by Ilizarov external fixator could be carried out if obvious limb discrepancy occurred.
5. pay attention to primary vascular and nerve injuries of the affected foot, and avoid iatrogenic injuries during the operation and stretching process.
6. the combined utilization of internal and external fixators could reduce surgical trauma.

## 4 Conclusions

In conclusion, although the design and the skill of surgery have high demands on doctors, the staged corrective surgical strategy of Ilizarov external fixation technology combined with limited osteotomy can effectively and safely correct stiff ankle deformities with cicatricial contracture of soft tissues, preserve the length of the affected lower limb, and reduce the demand for implants. This therapeutic strategy can also prevent surgical complications that frequently challenge the conventional one-stage corrective osteotomy plan, such as soft tissue necrosis, incision infection, and neurovascular bundle injury.

## Acknowledgments

We thank the patient for providing consent for this case report.

## Author contributions

**Conceptualization:** LuPing Liu.

**Data curation:** LeiJie Chen.

**Resources:** QiHui Duan.

**Visualization:** Zhou Liu.

**Writing – original draft:** Jie Zhang, YongMei Li.

**Writing – review & editing:** LuPing Liu, Bo Pu.
